# Recent advances in research on novel therapeutic mechanisms and strategies for exosome-based treatment of ischemic stroke

**DOI:** 10.3389/fphar.2026.1930673

**Published:** 2026-09-22

**Authors:** Tairan Liu, Hanlin Cao, Xi Chen, Jiaxin Huang, Qiao Zhang, Mingze Sun, Wen Zeng, Yu Su

**Affiliations:** 1 Department of Cell Biology, Third Military Medical University, Chongqing, China; 2 Department of Neurology, Southwest Hospital, Third Military Medical University (Army Medical University), Chongqing, China; 3 Department of Pain and Rehabilitation, Xinqiao Hospital, Army Medical University, Chongqing, China; 4 Jinfeng Laboratory, Chongqing, China

**Keywords:** blood–brain barrier, cerebral ischemia-reperfusion injury, engineered exosomes, exosomes, ischemic stroke, mesenchymal stem cells, miRNA, targeted delivery

## Abstract

Ischemic stroke (IS) accounts for over 80% of all stroke cases, featuring high disability, mortality, and recurrence rates. Conventional intravenous thrombolysis and mechanical thrombectomy are limited by narrow therapeutic windows, reperfusion injury, and the blood–brain barrier (BBB), which impedes the entry of neuroprotective agents into the brain parenchyma. Exosomes (Exos) are cell-secreted nanoscale extracellular vesicles (EVs) that carry miRNAs, proteins, and lipids, with low immunogenicity, BBB permeability, and ease of genetic modification, making them promising cell-free therapeutic agents for central nervous system (CNS) disorders. Exos alleviate cerebral ischemia-reperfusion injury by suppressing multiple forms of programmed cell death, oxidative stress, and neuroinflammation, repairing the BBB, and facilitating angiogenesis and neurogenesis. Genetic engineering, surface peptide modification, and drug loading further enhance brain-targeted exosomal enrichment. This review systematically summarises Exos biogenesis, multi-pathway neural repair mechanisms mediated by exosomal miRNAs, engineered delivery strategies, translational bottlenecks, and future research directions, aiming to provide theoretical support for the clinical transformation of Exos therapy for IS.

## 1 Introduction

Ischemic stroke (IS), characterized by intracranial vascular occlusion, is one of the leading causes of disability and mortality worldwide (Benjamin et al., 2017; Campbell et al., 2019a; Zhang H. et al., 2023). Following vascular occlusion, microglia, astrocytes, and neurons within the lesion exhibit morphological abnormalities, accompanied by degenerative changes in neuronal cell bodies and axons (Nguyen et al., 2016; Park et al., 2013), which in turn induce neuroinflammation, disruption of the blood-brain barrier (BBB), and massive neuronal death, ultimately leading to cerebral oedema and persistent neurological deficits (Macrez et al., 2011; Steliga et al., 2020; Datta et al., 2020). Even after reperfusion is achieved, ischaemia-reperfusion injury continues to damage neurons, hindering patients’ long-term functional recovery (Lan et al., 2024).

Currently, the preferred interventions for acute IS are intravenous thrombolysis and mechanical thrombectomy within the therapeutic time window (Yogendrakumar et al., 2024). Under current protocols, the time window for intravenous thrombolysis is 4.5 h from symptom onset, while mechanical thrombectomy is indicated within 24 h for patients with large-vessel occlusion (Ho and Powers, 2025); screening for eligible patients can extend the thrombolysis window to 9 h, and the new thrombolytic agent tenapeptase can further extend it to 24 h (Campbell et al., 2019b; Xiong et al., 2024). However, due to strict patient inclusion and exclusion criteria (including imaging-confirmed acute cerebral ischemia within the therapeutic time-window; exclusion of baseline intracranial hemorrhage, high-volume infarction and contraindications to intravenous agents), and a high risk of hemorrhagic transformation closely associated with BBB disruption—while BBB opening permits nanoparticle-based drug delivery to ischemic lesions, excessive barrier breakdown increases bleeding risk and disqualifies vulnerable patients from acute intervention, only a small number of patients can benefit promptly. At the same time, most survivors are left with long-term sequelae such as hemiplegia, cognitive impairment, and aphasia. The core bottleneck lies in the BBB and the blood-cerebrospinal fluid barrier (BCSFB), which prevent drugs from entering the brain, and in the lack of targeted drugs capable of simultaneously regulating inflammation, vascular damage, and neurological injury (Ishii et al., 2012; Doyle et al., 2015; Cuartero et al., 2019). Thrombolysis and interventional therapies also carry serious complications, such as intracranial haemorrhage and reperfusion injury (Zerna et al., 2018; Powers et al., 2019); therefore, there is an urgent need to develop novel formulations that can cross the BBB and provide both acute neuroprotection and long-term neural repair.

Exosomes (Exos), extracellular vesicles (EVs) with a diameter of 40–160 nm, have gradually garnered attention since their formal designation in 1987 (Pan and Johnstone, 1983; Kalluri and LeBleu, 2020). Various human cell types secrete them, and they are stably present in body fluids such as cerebrospinal fluid and blood (Lasser et al., 2011). As natural biological nanocarriers, Exos carry signaling molecules, including proteins and nucleic acids, to mediate cellular communication. Compared to synthetic nanomaterials, they offer the advantages of low immunogenicity and high stability (Kalluri and LeBleu, 2020) and possess the innate ability to cross the BBB. Previous studies have confirmed that Exos derived from neural stem cells can cross the BBB via endocytosis by endothelial cells (Joshi and Zuhorn, 2021) and exert neuroprotective effects by regulating inflammation, apoptosis, and neurovascular regeneration in IS models (Venkat et al., 2018). However, the brain-tissue targeting efficiency of native Exos is relatively low, and various engineering modification strategies are being employed to optimise their brain enrichment (Khan et al., 2021). This article systematically summarises the molecular mechanisms by which Exos mediate the repair of ischaemic brain injury, as well as targeted delivery systems, and analyses their advantages for clinical translation and existing challenges, providing a theoretical framework for cell-free therapies in IS.

## 2 Composition and biogenesis of exosomes

### 2.1 Exosomes structure and active components

Exos are spherical bilayer lipid vesicles with a diameter of 30–150 nm and belong to the core subtype of EVs, distinct from microvesicles and apoptotic bodies (Shao et al., 2020). The vesicle membrane is rich in the tetratransmembrane protein family (CD9, CD63, CD81), heat shock proteins (HSP70 and HSP90), proteins involved in sorting and endosomal transport—such as TSG100 and Alix—as well as the sphingolipid ceramide (Yanez-Mo et al., 2015; Thery et al., 1999; Willms et al., 2018). Membrane lipids are rich in cholesterol, sphingolipids, and phosphatidylserine; the bilayer membrane structure not only maintains the stability of Exos in bodily fluids such as blood and cerebrospinal fluid but also mediates fusion between vesicles and target cell membranes, thereby enhancing the efficiency of content delivery (Skotland et al., 2019).

The lumen of Exos contains a variety of bioactive molecules, including DNA, mRNA, circRNA, lncRNA, microRNA (miRNA), cytokines, and metabolites (Lasser, 2015). Among these, miRNAs are key functional mediators relevant to IS pathology: only 20–24 nt in length and non-coding, they bind to Ago2 to form the RISC complex, post-transcriptionally inhibit the translation of target mRNAs, and participate in the regulation of pathways such as inflammation, apoptosis, and angiogenesis (Mantel et al., 2016; Abdul Muneer, 2016). Ribonucleases readily degrade free miRNAs and cannot be stably transported between cells; the lipid bilayer of Exos can insulate them from enzymes, protecting miRNAs and ensuring their intact transport to distant target cells, thereby enabling long-distance cellular communication (Zheng et al., 2020; Tran, 2016).

Brain tissue is rich in circRNAs; brain-derived exosomal circRNAs can serve as non-invasive diagnostic biomarkers for acute IS, offering new insights into early stroke screening (Jiang et al., 2024). Of particular interest for this review, thanks to their unique lipid-protein composite structure, Exos possess the innate ability to cross the BBB, thereby bypassing it as a drug-delivery barrier and serving as an ideal carrier for targeted drug delivery in IS (Abdelsalam et al., 2023; Banks et al., 2020). Exosomal surface membrane proteins can be artificially modified and engineered to carry targeted peptides, thereby further enhancing enrichment efficiency in brain tissue and laying the foundation for the development of engineered delivery systems (Yang et al., 2025a).

### 2.2 Exosomes biogenesis and secretion pathways

Exos biogenesis begins with plasma membrane endocytosis, producing early endosomes that mature into late endosomes. Late endosomes acquire intraluminal vesicles (ILVs) through inward membrane budding and are then defined as multivesicular bodies (MVBs). This process is regulated by Rab27a, ESCRT, ALIX, and TSG101 (Song L. et al., 2019; Lai et al., 2022; van Niel et al., 2018). Mature multivesicular bodies (MVBs) follow two metabolic pathways: transport along microtubules to the cell membrane, where they fuse and release Exos; or fusion with lysosomes, leading to degradation (Mincheva-Nilsson et al., 2016; Kalluri, 2016). Virtually all cell types—including stem cells, microglia, and astrocytes—can secrete Exos, which are widely present in body fluids such as blood and cerebrospinal fluid (Doyle and Wang, 2019; Song Y. et al., 2019). Furthermore, the composition and function of Exos are determined by the type of donor cell and the microenvironment (Kwak et al., 2022; Ahn et al., 2021).

Once released, Exos facilitate intercellular communication through three mechanisms: direct membrane ligand-receptor interactions, indirect regulation via soluble ligands, and endocytic uptake of cargo by target cells, thereby mediating biological effects such as cell proliferation and immune regulation (Mehdizadeh et al., 2025). From the perspective of stroke-targeted delivery, thanks to their complete biosynthetic pathway, Exos possess the innate ability to cross the BBB and serve as targeted delivery vehicles for IS (Terstappen et al., 2021) (Figure 1).

**FIGURE 1:**
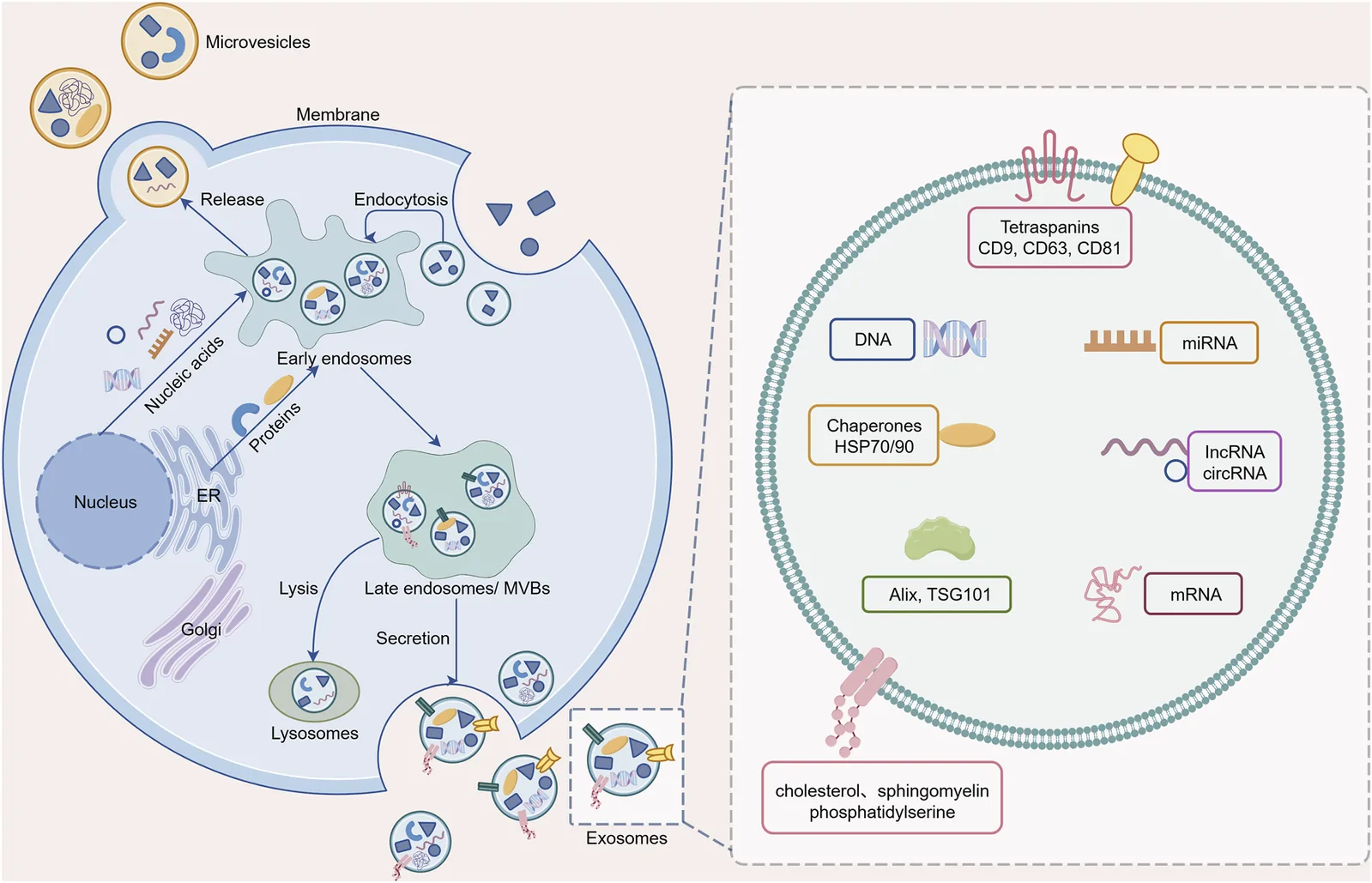
Exos composition and a schematic diagram of biological development. Created with figdraw.com (confirmation number: ASSRPa5af0).

## 3 Pathological mechanisms of stroke and the regulatory roles of endogenous and exogenous exosomes

### 3.1 Pathological mechanisms of stroke

Following vascular occlusion, the supply of glucose and oxygen to neurons is interrupted. Disrupted energy metabolism leads to the massive production of reactive oxygen species (ROS) by mitochondria, inducing oxidative stress damage (Andrabi et al., 2020). Energy depletion disrupts the ionic balance of the cell membrane, resulting in sustained intracellular Ca^2+^ overload. This triggers massive glutamate release and excessive activation of NMDAR receptors, ultimately leading to excitotoxicity in neurons (Wu and Tymianski, 2018; Choi et al., 1988; Garthwaite et al., 1992; Olney, , 1969). NMDARs exhibit bidirectional regulatory effects: the GluN2B subtype mediates damage, while the GluN2A subtype exerts a protective effect; extrasynaptic NMDARs further exacerbate apoptosis (Wu and Tymianski, 2018; Zhu et al., 2018).

Cerebral ischemia-reperfusion is a complex injury process involving the synergistic interaction of multiple pathways (Yu et al., 2023). Following reperfusion and restoration of blood supply, an imbalance in glutamate metabolism and excessive nitric oxide production, together with free radicals, damage cell membrane lipids and nucleic acid structures (Wang Y. et al., 2022; Terpolilli et al., 2012). Calcium overload acts as a central hub, simultaneously activating multiple damage pathways: early autophagy clears damaged organelles and exerts a protective effect, whereas persistent calcium overload and oxidative stress induce excessive autophagy and endoplasmic reticulum stress, ultimately triggering the apoptotic programme (Huang et al., 2019; Jiang et al., 2023).

Necrotic cells release DAMPs, which activate microglia in the brain and induce infiltration of neutrophils and monocytes. The massive release of pro-inflammatory cytokines damages the microvascular endothelium and disrupts the integrity of the BBB, creating a cycle of amplified inflammation (Shi et al., 2016; Henrot et al., 2023; Chen H. et al., 2020). During the reperfusion phase, multiple forms of programmed cell death are simultaneously activated: the mitochondrial pathway mediates apoptosis (Datta et al., 2020); the NLRP3 inflammasome drives pyroptosis, which is highly coupled with inflammation (Wang L. et al., 2022); lipid peroxidation and iron metabolism disorders induce ferroptosis (Tuo et al., 2022); and necrotic apoptosis and pan-apoptosis further exacerbate the expansion of the infarct zone. Current intravenous thrombolysis and mechanical thrombectomy can only restore vascular patency; they are subject to a strict time window and cannot simultaneously inhibit the multiple damage cascades mentioned above. Traditional small-molecule and peptide drugs struggle to penetrate the intact BBB, resulting in limited clinical translation. All of the aforementioned pathological processes can be regulated by nucleic acids and protein signals carried by Exos, which serve as core targets for their neuroprotective effects.

Multiple studies have now confirmed that Exos from various sources can regulate the process of cerebral ischemic injury through multicellular and cross-organ pathways: (1)Pre-stroke exercise adaptation promotes the secretion of exosomal miR-484 by skeletal muscle; this miRNA specifically inhibits ACSL4 and upregulates the SLC7A11/GPX4 pathway, significantly suppressing neuronal ferroptosis, reducing infarct volume, and improving neurological function, thereby revealing a novel cross-axis protective mechanism involving skeletal muscle–Exos–miRNA–brain-distant organs (Huang et al., 2024). (2) Ischemic stress can upregulate Rab27a expression in brain tissue, promoting the secretion of functional Exos; Rab27a deficiency significantly depletes protective Exos in the brain, exacerbates endothelial oxidative stress and apoptosis, disrupts cerebral vascular homeostasis, and worsens cerebral infarction. Exos from wild-type brain tissue can reverse this damage. In contrast, Exos derived from Rab27a-deficient cells exhibit significantly reduced reparative activity (Ma et al., 2022). (3) Hypoxic vascular smooth muscle cell Exos are enriched with Src protein, which can mediate the polarization of microglia toward the pro-inflammatory M1 phenotype and exacerbate inflammatory damage caused by stroke; inhibition of Src with dasatinib can reverse the M1/M2 imbalance in microglia, reduce neuronal apoptosis, and improve neurological function (Zhang et al., 2024) (Figure 2).

**FIGURE 2:**
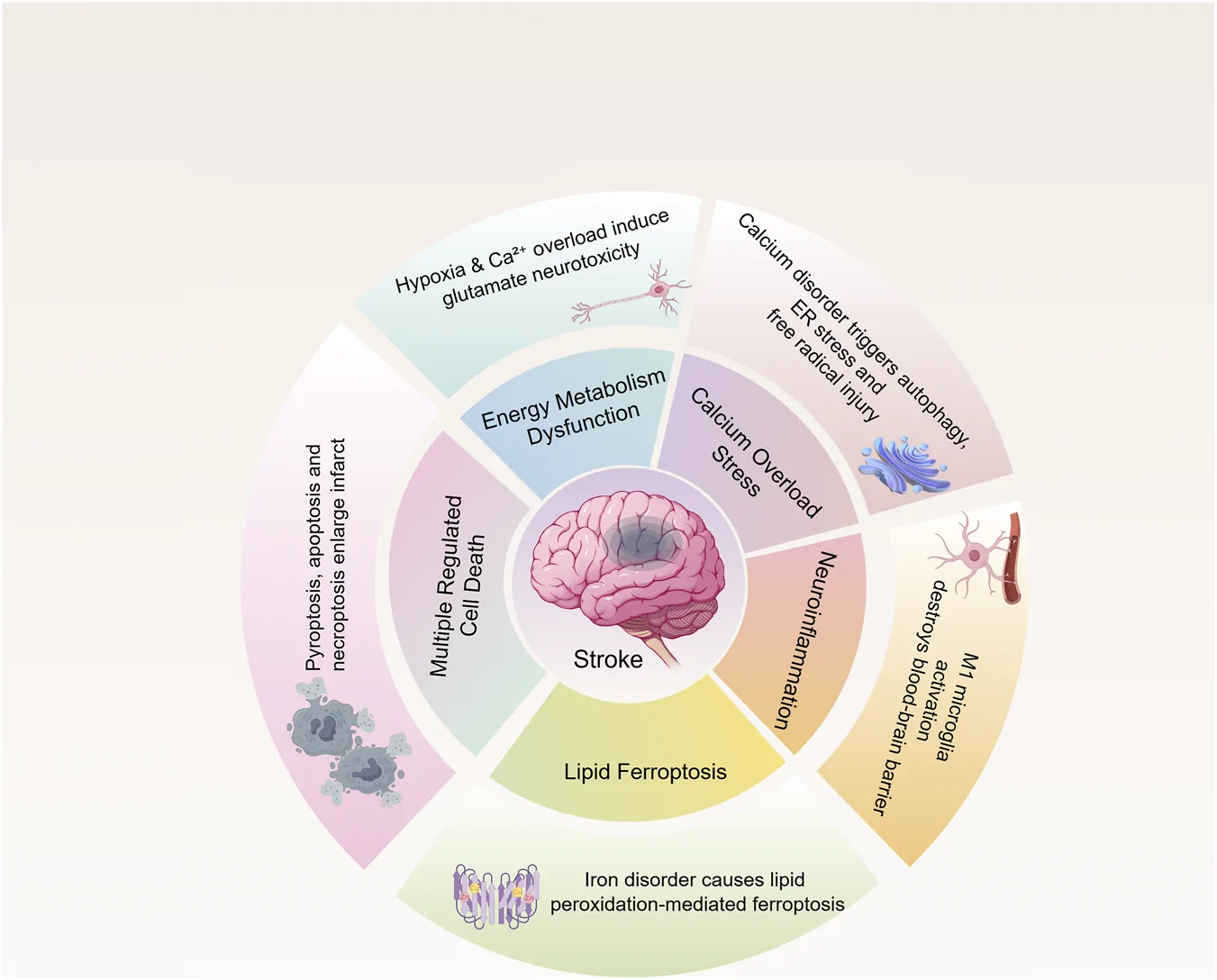
Double-Layer Donut Chart of Five Core Pathological Pathways in Cerebral Ischemia-Reperfusion Injury. Created with figdraw.com (confirmation number: IORIR9c11c).

### 3.2 The dual regulatory role of endogenous exosomes in ischemic stroke

Neurons, microglia, astrocytes, and cerebral vascular endothelial cells can all stably secrete endogenous Exos. The ischaemic-hypoxic microenvironment reshapes the multivesicular body-sorting pathway, altering Exo release levels and their internal nucleic acid and protein cargo, thereby causing Exos derived from different cell types and at different stages of the disease process to exert either damaging or protective effects.

In the early stages of ischaemia, activated M1-type microglia release injurious Exos that trigger inflammatory cascades via the pro-inflammatory miRNAs they carry, thereby disrupting the BBB and accelerating neuronal apoptosis in the penumbra. Their pro-inflammatory and injurious effects have been validated in the MCAO model. The research team led by Yan Guangmei and Yin Wei discovered that HK2-mediated phosphorylation of nSMase1 promotes astrocyte Exos biogenesis, which in turn disrupts the BBB and exacerbates acute ischemic brain injury (Chen et al., 2025). In patients with atherosclerotic stroke, the miRNA Novel3 is significantly upregulated in plasma Exos derived from foam cells. This molecule targets intracerebral myeloid cells, inhibits the STRN-mediated PI3K-AKT pathway, exacerbates neuroinflammation and ferroptosis, and, when inhibited, alleviates ischaemic injury, making it a potential intervention target for atherosclerosis-related stroke (Qin C. et al., 2024). Under ischemic stimulation, macrophage Exos highly express THBS1; this protein binds to OTUD5 and mediates the ubiquitination of GPX4, inducing ferroptosis in cerebral vascular endothelial cells and disrupting the BBB. The small molecule salicylic acid B can block the THBS1 pathway and alleviate cerebral ischaemic-reperfusion injury (Liu C. et al., 2025). In patients with atherosclerotic large-artery stroke, Exos secreted by macrophages in atherosclerotic plaques show upregulated miR-30c-2-3p expression. This molecule targets microglia in the brain and inhibits the SMAD2 pathway, thereby exacerbating neuroinflammation and cerebral ischaemic injury and establishing a mechanism linking peripheral arterial plaques to brain tissue damage (Tang et al., 2024). Following IS, peripheral blood Exos show high HABP2 expression. This protein inhibits astrocyte autophagy by activating the PAR1/PI3K/AKT/mTOR pathway, excessively activates neuroinflammation, and induces neuronal apoptosis, thereby exacerbating cerebral infarction damage; it represents a key deleterious regulatory mechanism mediating secondary damage in IS (Luo et al., 2023).

During the mid-to-late stages of stroke, endogenous brain cells switch to secreting reparative Exos that facilitate compensatory repair of brain tissue by regulating glial polarization and promoting angiogenesis. Clinical samples confirm that the peripheral exosomal circCNOT6L is significantly downregulated in stroke patients, and its expression is negatively correlated with infarct volume and NIHSS score. *In vitro* experiments confirm that this circRNA can bind to miR-99a-5p via a sponge-like mechanism, upregulate SERPINE1, and antagonise astrocyte apoptosis (He et al., 2023). Targeted inhibition of PDE1-B in microglia can reshape the M1/M2 balance, regulate the release of protective Exos, and alleviate neuronal ischemic injury (Zang et al., 2020). Brain-derived exosomal nucleic acid molecules in circulation and cerebrospinal fluid can objectively reflect the extent of stroke damage and serve as non-invasive biomarkers for disease stratification and long-term prognosis assessment, demonstrating clinical translational value.

### 3.3 Potential and limitations of exogenous exosomes intervention in ischemic-reperfusion injury

Exos isolated *in vitro* from stem cells are the mainstream cell-free therapeutic carriers in the field of stroke, with research systems involving Exos derived from bone marrow mesenchymal stem cells and neural stem cells being the most well-established (Roy et al., 2025; Yang Y. et al., 2025; Sun Y. et al., 2025). Compared to stem cell transplantation, Exos, as cell-free biological agents, can effectively avoid safety risks associated with allogeneic transplantation, such as immune rejection, ectopic tissue proliferation, and microvascular embolism. Their natural lipid membrane structure allows them to penetrate the BBB via receptor-mediated endocytosis by cerebral vascular endothelial cells, where they accumulate specifically at ischaemic sites. Through synergistic multi-pathway mechanisms, they inhibit oxidative stress, neuroinflammation, and programmed cell death, thereby consistently reducing infarct volume and improving motor and cognitive deficits in animal models of ischaemia (Sun Y. et al., 2025).

Natural, unmodified Exos still have significant limitations in terms of delivery efficiency: following systemic intravenous administration, the vast majority of vesicles are captured and cleared by the liver and spleen’s reticuloendothelial system, resulting in extremely low levels of effective accumulation in brain tissue (Choi et al., 2021; Smyth et al., 2015); batch-to-batch variations in stem cell culture can easily lead to fluctuations in the protein and nucleic acid cargo components within Exos, and the inherent limitations of natural vesicles’ drug-loading capacity constrain the therapeutic efficacy of single-component formulations (Yang Y. et al., 2025).

To address these limitations, existing studies have engineered Exos by surface-conjugating brain-targeting peptides, genetically engineering membrane proteins, and co-loading small-molecule drugs, thereby enhancing brain-targeted accumulation efficiency and strengthening multifaceted neuroprotective effects. The following sections will systematically review the molecular regulatory mechanisms by which Exos counteract cerebral ischaemia-reperfusion injury and categorise progress in targeted modification strategies. Representative studies focusing on native exosomes for cerebral ischaemia‐reperfusion injury are listed in Table 1.

**TABLE 1:** Summary of representative studies of native Exos for cerebral ischaemia-reperfusion injury.

Exosomes source	Key cargo	Target signalling pathway	Pathophysiological role	Main outcomes	References
Foam cells (atherosclerotic plaque)	miRNA Novel-3	PI3K-AKT-mTOR	Pro-inflammatory, ferroptosis induction	Elevated in LAA-AIS; blocking Novel-3 relieves ischaemic brain injury	(Qin et al., 2024a)
Hypoxic vascular smooth muscle cells (VSMCs)	Src protein	Src-mediated microglial polarization	Pro-inflammatory, M1 polarization	Hypoxic VSMC-exos promote M1 microglia; Src inhibitor dasatinib suppresses M1 and improves function	(Zhang et al., 2024)
Macrophages (atherosclerotic plaque)	THBS1	THBS1-OTUD5-GPX4 axis	Endothelial ferroptosis, BBB disruption	Elevated in AIS Exos; silencing THBS1 relieves cerebral I/R injury	(Liu et al., 2025a)
Foam cells (atherosclerotic plaque)	miR-30c-2-3p	TGF-β/SMAD2	Pro-inflammatory microglial polarization	Elevated in LAA-AIS; inhibiting miR-30c-2-3p alleviates neuroinflammation	(Tang et al., 2024)
Neural cells	lncRNA OIP5-AS1	TXNIP/NLRP3 pyroptosis pathway	Anti-pyroptosis, neuroprotection	Downregulated in AIS Exos; overexpressing OIP5-AS1 alleviates cerebral I/R injury	(Li et al., 2024)
Brain microvascular endothelial cells	Multiple cargoes	PDGF-PDGFRβ, Ang1/Ang2-Tie2	BBB protection	EC-Exo reduces BBB leakage, improves motor function after I/R injury	(Sun et al., 2025b)
Skeletal muscle (exercise preconditioning)	miR-484	ACSL4/SLC7A11-GPX4	Anti-ferroptosis, cross-organ protection	Reduce infarct volume and improve long-term neurological function via miR-484	(Huang et al., 2024)

## 4 Mechanisms by which exos regulate ischemia-reperfusion repair

Exogenous stem cells, glial cells, pretreated and modified Exos, and engineered Exos can deliver functional cargoes such as proteins, circRNA, miRNA, and lncRNA to target and intervene at every stage of ischaemia-reperfusion injury. These mechanisms involve cell-specific pathways, including inhibiting programmed cell death, providing antioxidant effects, regulating glial polarisation, repairing the BBB, and promoting vascular and neural remodelling, thereby forming a multi-level synergistic protective network. The specific mechanisms of each regulatory pathway are as follows.

### 4.1 Inhibition of programmed cell death

Ferroptosis is a core mechanism of ischaemic neuronal damage; various natural and engineered stem cell-derived Exos can target and modulate key ferroptosis pathways to exert protective effects. Zhao Jing’s team demonstrated that human umbilical cord mesenchymal stem cell Exos inhibit the ARRDC3–DRP1 pathway by delivering the CRYAB protein, thereby stabilising mitochondrial dynamics, alleviating neuronal ferroptosis, and improving neurological function in MCAO mice, providing a novel protein-mediated protective target for ischaemia-reperfusion injury (ji et al., 2026). Adipose-derived stem cell Exos exhibit a dual anti-ferroptosis effect: on the one hand, they activate the FXR2/ATF3/SLC7A11 pathway to alleviate ferroptosis in M2 microglia and indirectly improve neurological damage (Wang Y. et al., 2024); following intranasal administration, they accumulate in the brain parenchyma and, by carrying miR-760-3p to silence the core ferroptosis gene CHAC1, directly block the neuronal ferroptosis process (Wang Y. et al., 2023). Genetically modified Exos can enhance anti-ferroptotic efficacy; Exos from bone marrow mesenchymal stem cells overexpressing FNDC5 are enriched in irisin, which inhibits the YAP/EGR1/ACSL4 signaling axis, thereby alleviating mitochondrial oxidative damage, improving neuronal survival rates, and reducing cerebral infarct volume (Fang et al., 2025). Circular RNAs are also key mediators of Exos-regulated ferroptosis. The circBBS2 circular RNA in umbilical cord mesenchymal stem cell Exos acts as an endogenous ceRNA sponge, binding miR-494 and thereby upregulating the ferroptosis inhibitor SLC7A11, effectively alleviating cerebral ischaemic-hypoxic injury in rats (Hong et al., 2023).

In addition to ferroptosis, Exos can block the NLRP3 inflammasome-dependent pyroptosis pathway. Exos from M2-type microglia carry the lncRNA OIP5-AS1, which recruits the ubiquitin ligase ITCH to mediate the ubiquitin-mediated degradation of TXNIP, thereby inhibiting downstream neuronal pyroptosis (Li et al., 2024). Plasma Exos pretreated with melatonin specifically inhibit the TLR4/NF-κB pathway, block NLRP3 activation, alleviate microglial and neuronal pyroptosis, reduce infarct volume, and restore neural function (Wang K. et al., 2020) (Figure 3).

**FIGURE 3:**
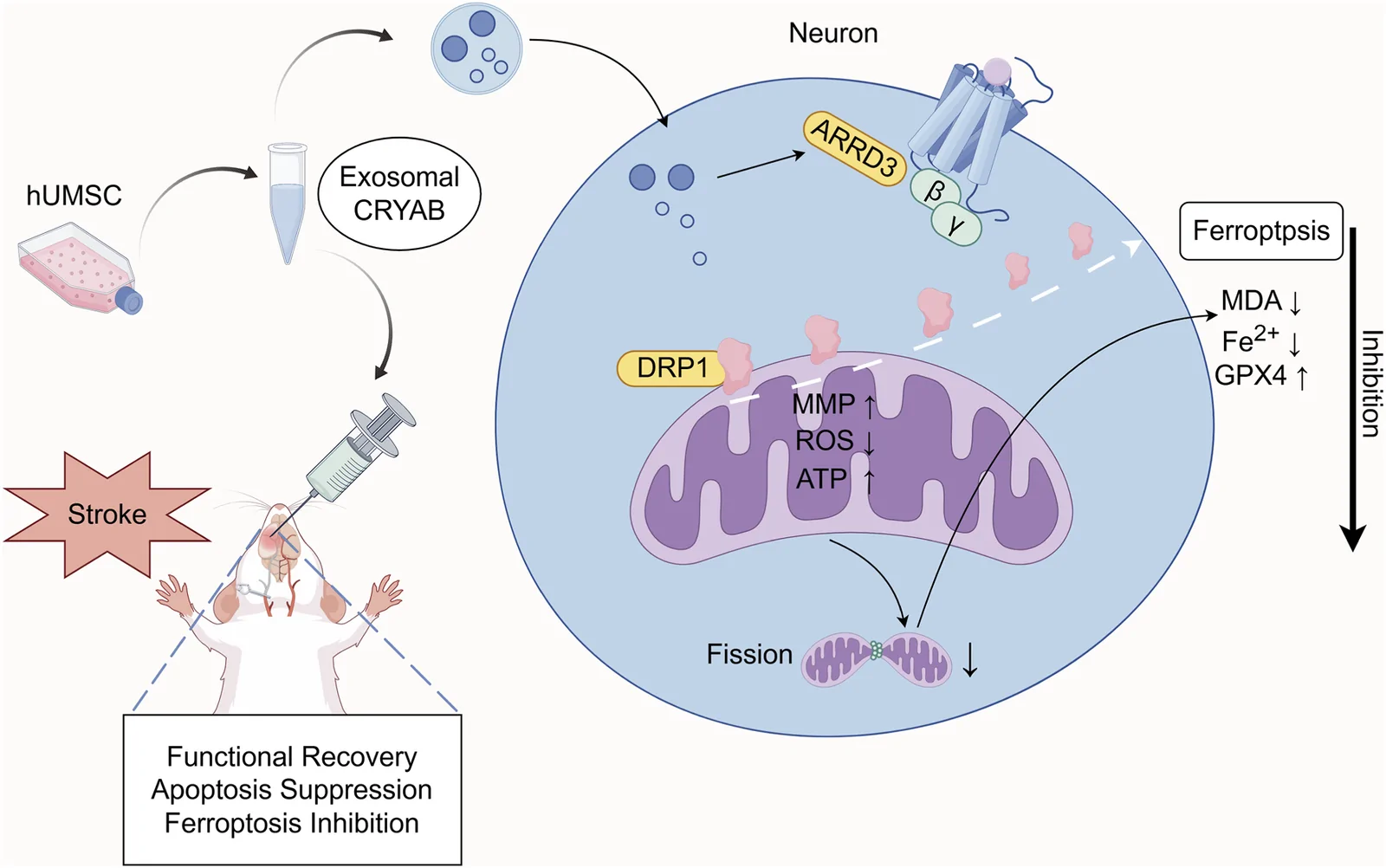
Intranasally administered hUMSC-derived Exos modulate the CRYAB–ARRDC3–Drp1 axis, alleviating mitochondrial dysfunction and ferroptosis, enhancing neuronal survival, reducing oxidative stress, and promoting functional recovery from ischaemia-reperfusion injury, thereby offering a promising therapeutic strategy for IS (ji et al., 2026). Created with figdraw.com (confirmation number: YRSPT666aa). Abbreviations: hUMSC, human umbilical cord mesenchymal stem cell; ARRDC3, arrestin domain-containing 3; DRP1, dynamin-related protein 1; MMP, mitochondrial membrane potential; ROS, reactive oxygen species; MDA, malondialdehyde; GPX4, glutathione peroxidase four. Adapted from Ji et al., 2026.

### 4.2 Alleviating oxidative stress and improving mitochondrial function

Mitochondrial damage and excessive accumulation of ROS are the core triggers of ischaemic oxidative stress. Exos derived from various cell types can restore redox balance through multiple pathways, including the regulation of mitochondrial autophagy, antioxidant pathways, and epigenetic modifications. Exos derived from human neural stem cells activate the PINK1/Parkin pathway to induce mitochondrial autophagy, clear damaged mitochondria, reduce ROS accumulation, inhibit neuronal apoptosis, and alleviate brain damage in MCAO rats (Zhao M. et al., 2025). Electroporation-loaded engineered neural progenitor cell Exos containing miR-100-5p specifically silence NOX4, reduce oxidative stress levels in brain tissue, and simultaneously protect microvascular endothelium and promote angiogenesis (Zhao X. et al., 2025); Combined administration of Exos from neural progenitor cells and endothelial progenitor cells overexpressing miR-210 and miR-126 produces a synergistic protective effect, inhibiting Nox2-mediated oxidative stress, activating the BDNF/TrkB pathway, repairing neuronal dendritic spines, and reducing apoptosis (Xu et al., 2023). Exos from IL-4-induced M2 macrophages improve the viability of hypoxic neurons by activating the Nrf2/HO-1 antioxidant axis, thereby increasing SOD activity and reducing MDA and ROS accumulation; this protective effect can be completely blocked by the Nrf2 inhibitor ML385 (Fang et al., 2025).

Exos from endothelial progenitor cells deliver miR-210 to improve mitochondrial function specifically; upregulation of this miRNA further amplifies antioxidant and anti-apoptotic effects (Yerrapragada et al., 2022). Exos from bone marrow mesenchymal stem cells carry KLF4, which binds to the lncRNA-ZFAS1 promoter to inhibit Drp1 m6A methylation, thereby stabilizing mitochondrial dynamics and alleviating oxidative stress-induced damage (Wang QS. et al., 2023). The miR-133a-3p contained within these Exos specifically targets and inhibits DAPK2, thereby activating the Akt/mTOR pathway to prevent excessive autophagy-induced apoptosis in neurons (Yang et al., 2023). miR-22-3p in adipose-derived stem cell Exos silences KDM6B, downregulating the BMP2/BMF pathway and reducing neuronal apoptosis (Zhang et al., 2021); Bone marrow mesenchymal stem cells (BMSCs) Exos containing the lncRNA KLF3-AS1 sequester miR-206, upregulate USP22 to stabilise Sirt1, and simultaneously inhibit oxidative stress, inflammation, and apoptosis (Xie et al., 2023). Astrocyte Exos containing miR-190b specifically silence Atg7, thereby blocking ischemia-induced excessive neuronal autophagy and secondary inflammation (Pei et al., 2020). Treadmill exercise combined with BMSC Exos synergistically inhibits the JNK1/c-Jun pathway, reducing neuronal apoptosis and promoting axonal and synaptic regeneration (Pei et al., 2020); human amniotic epithelial cell Exos upregulate FoxM1, simultaneously alleviating oxidative stress in neurons and microglia and suppressing inflammatory apoptosis (Wang et al., 2025). Furthermore, miR-124 in BMSC Exos can upregulate the astrocyte glutamate transporter GLT-1, thereby alleviating ischemic glutamate excitotoxicity and indirectly mitigating oxidative stress-induced cascade damage (Huang et al., 2023) (Figure 4).

**FIGURE 4:**
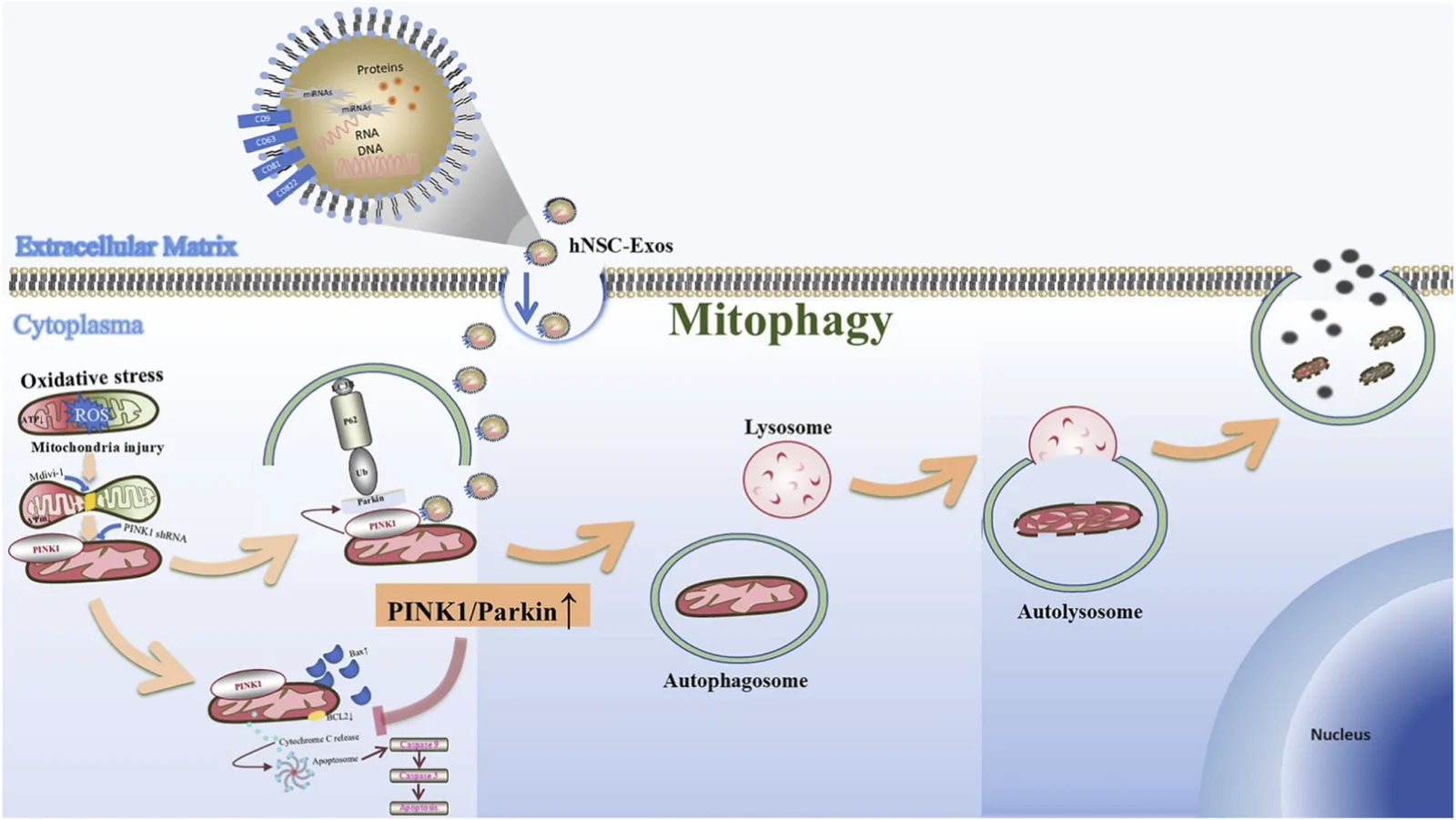
Human neural stem cell-derived Exos (hNSC-Exos) alleviate OS-induced neuronal damage by regulating the PINK1/Parkin pathway (Zhao M. et al., 2025). After internalization by neurons, hNSC-Exos upregulate the PINK1/Parkin pathway, which labels damaged mitochondria via p62/ubiquitin. This promotes autophagosome engulfment of impaired mitochondria and subsequent fusion with lysosomes to form autolysosomes, thereby clearing damaged mitochondria and reducing ROS. Concurrently, hNSC-Exos suppress the Bax/cytochrome c/Caspase apoptotic cascade, decreasing neuronal apoptosis. The mitophagy inhibitor Mdivi-1 and PINK1 knockdown abolish these protective effects.

### 4.3 Regulating microglia polarization and inhibiting neuroinflammation

An imbalance in M1/M2 microglial polarization is a key driver of amplified inflammation in stroke; Exos from different sources and those subjected to pretreatment can bidirectionally regulate microglial phenotypes, thereby reshaping the intracerebral immune microenvironment. Wang Fengshan and Zhang Xinke demonstrated that hypoxia-induced neural stem cell Exos highly express miR-125a-5p, which specifically inhibits the TLR4/NF-κB pathway, promoting the conversion of microglia to the anti-inflammatory M2 phenotype and suppressing local inflammatory infiltration (Liu Q. et al., 2025). In the same year, Zhao Jing’s team demonstrated that intranasal delivery of umbilical cord mesenchymal stem cell Exos can target ischemic lesions; by inhibiting the TREM1-p38 MAPK pathway via HMGB1, they induce an anti-inflammatory microglial phenotype and reduce neuronal apoptosis (Zhang Z. et al., 2025). Pre-treatment with natural medicines can optimise the anti-inflammatory function of Exos: ginkgolide B upregulates exosomal miR-299a-3p to inhibit TRIL and activate the PI3K/AKT pathway, thereby alleviating oxidative inflammation in microglia and improving post-stroke cognitive impairment (Zhao D. et al., 2026). Pre-treatment of astrocyte Exos with berberine results in high expression of miR-182-5p, which specifically inhibits the Rac1 pathway, thereby downregulating pro-inflammatory factors such as IL-6, IL-1β, and TNF-α, demonstrating superior anti-inflammatory effects compared with Exos pre-treated with hypoxia alone (Ding et al., 2023).

Engineered targeted Exos can precisely block astrocyte-mediated inflammatory damage; EXO-RVG-SD208 delivers a TGF-β1 inhibitor, reverses neuroinflammation mediated by the TGF-β1/Smad2/3 pathway, and regulates the mTOR and BDNF pathways to promote synaptic repair (Zhao W. et al., 2026). Exos from LPS-pretreated macrophages can directly induce microglial polarization from M1 to M2, reducing infarct volume and improving neurological deficits in animals (Zheng et al., 2019). Exos from hypoxic cerebral microvascular endothelial cells are highly loaded with miR-3613-3p, which targets and activates RC3H1 to promote pro-inflammatory microglial polarization and exacerbate neuronal damage (Zhang M. et al., 2023); silencing this miRNA reverses vascular and neuronal damage. Human umbilical vein endothelial cell Exos upregulate the protective transcription factor KLF14, thereby inhibiting the release of multiple pro-inflammatory factors and alleviating ischemic brain injury (Qin J. et al., 2024). Exos from M2 macrophages are enriched in miR-330-5p, which inhibits the Syk/Stat3 pathway to reduce the secretion of inflammatory mediators; their anti-inflammatory effect is superior to that of Exos from resting M0 macrophages (Song et al., 2026). Exos from adipose-derived stem cells overexpressing miR-30d-5p block M1 microglial polarization by inhibiting autophagy, thereby alleviating damage during the acute phase of cerebral infarction (Jiang et al., 2018).

Neural stem cell Exos preferentially protect ischemic astrocytes and are suitable for intervention during the acute phase of stroke (Sun et al., 2019). miR-451a in plasma Exos derived from ischemic preconditioning inhibits the Rac1 pathway, thereby alleviating oxidative stress and neuronal apoptosis (Li H. et al., 2021). Exos from BMSCs overexpressing miR-138-5p specifically silence LCN2 in astrocytes, promoting glial cell survival and inhibiting inflammatory apoptosis (Deng et al., 2019). The lncRNA KLF3-AS1 in BMSC Exos regulates the Sphk1/NF-κB pathway via the YY1/MSI1 axis, thereby comprehensively inhibiting the ischaemia-reperfusion inflammatory cascade (Cao et al., 2024) (Figure 5).

**FIGURE 5:**
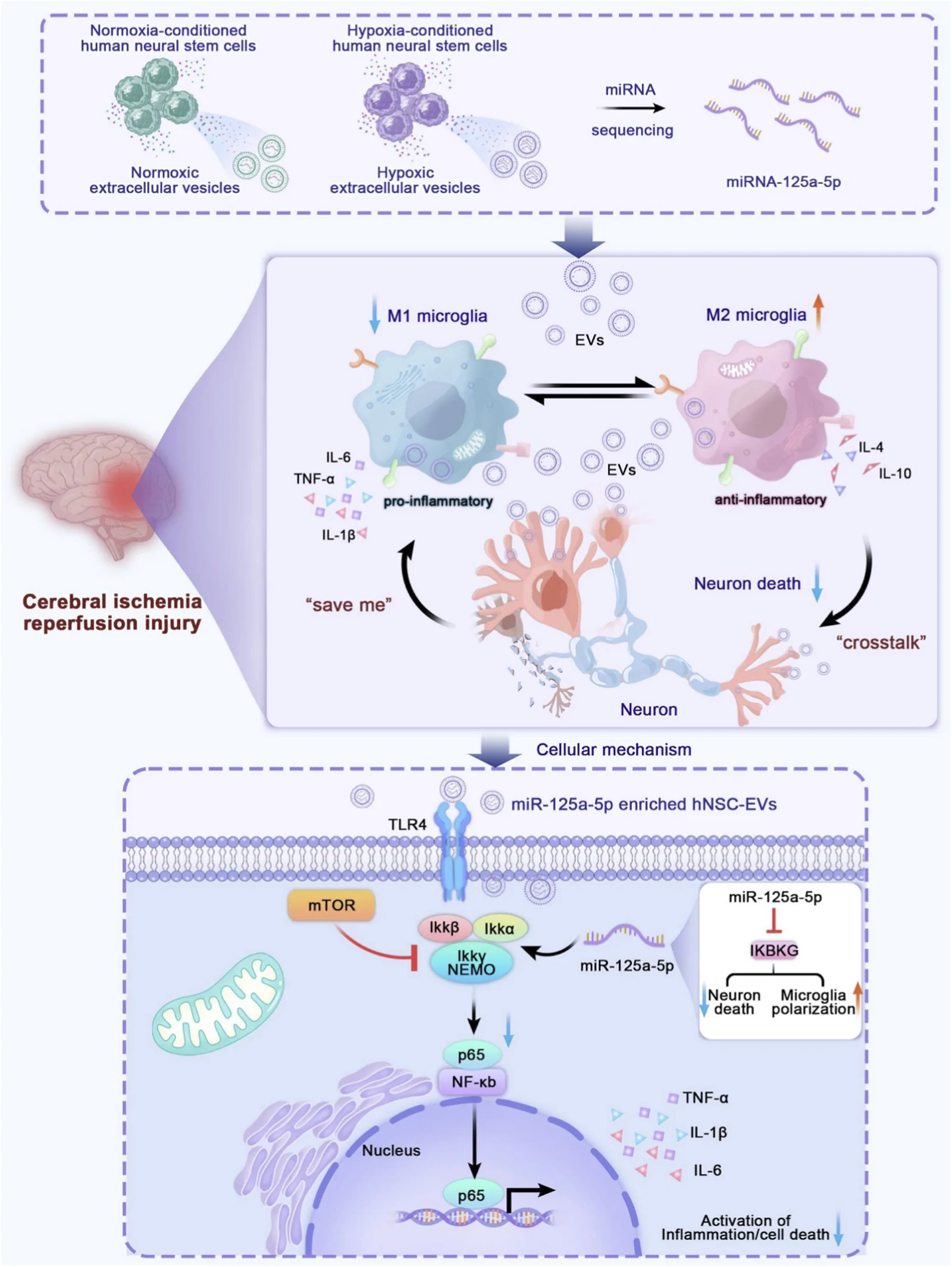
Scheme of hNSC-EV-mediated neural repair via miR-125a-5p delivery. Following MCAO, ischemic hNSCs release extracellular vesicles enriched in neural-repair-associated miRNAs. These EVs enter the circulation and are taken up by microglia and neurons, where they target the mTOR and TLR4/NF-κB pathways to promote neurogenesis and alleviate neuroinflammation. In particular, miR-125a-5p, enriched in ischemic hNSC-EVs, drives microglial polarization and rescues neuronal injury by directly targeting IKBKG (Liu Q. et al., 2025).

### 4.4 Repairing the blood-brain barrier and reducing vascular permeability

Damage to the BBB and activation of matrix metalloproteinases are key triggers for the development of vasogenic oedema and haemorrhage following ischaemia. Various Exos can upregulate tight junction proteins, protect vascular endothelial cells and pericytes, and restore vascular homeostasis. BMSC Exos, which are rich in miR-21a-5p, inhibit the TLR2/NF-κB pathway, downregulate MMP-2/9 activity, and upregulate the expression of tight junction proteins such as Claudin-5, Occludin, and ZO-1, thereby reducing BBB leakage and the transition to haemorrhage (Yang et al., 2026). Exos from cerebral microvascular endothelial cells activate the PDGF-PDGFRβ and Ang1/Ang2-Tie2 pathways, enhance endothelial-pericytic communication, repair the basement membrane and barrier structure, and improve neurobehavioral function in ischemic mice (Sun J. et al., 2025). Exos from the serum of healthy rats inhibit endothelial apoptosis by upregulating Bcl-2/Bax and simultaneously blocking barrier disruption mediated by excessive autophagy, thereby reducing infarct volume (Huang et al., 2022). In a diabetic context, BMSC-derived Exos downregulate miR-9 and activate the ABCA1/IGFR1 pathway, thereby independently regulating blood glucose levels to protect the BBB and promote white matter repair; overexpression of miR-9 completely counteracts this protective effect (Venkat et al., 2020).

Exos derived from cerebral microvascular endothelial cells can increase cerebral blood flow in ischemic regions and upregulate synaptic proteins to promote neural repair (Sun et al., 2022). miR-340-5p in Exos from the serum of healthy individuals specifically inhibits CD147, thereby promoting endothelial angiogenesis. In contrast, the expression of this molecule in peripheral Exos from stroke patients is significantly downregulated, thereby impairing their vascular protective capacity (Xu et al., 2022). Exos from CXCR4-overexpressing engineered BMSCs inhibit the Wnt-3a/β-catenin pathway, promote endothelial proliferation and luminal formation, antagonize endothelial apoptosis, and sustainably improve long-term neurological deficits (Li X. et al., 2020). ACE2-modified endothelial progenitor cell Exos are enriched for miR-17-5p, which specifically inhibits PTEN, thereby activating the PI3K/Akt pathway and alleviating post-ischemic endothelial senescence in aged mice, thereby enhancing microvascular density and local blood flow (Pan et al., 2023). HSP70 on the surface of plasma-derived Exos binds to endothelial TLR4 to facilitate cross-barrier transport; intracellular delivery of HSP70 scavenges ROS, stabilises mitochondria, and protects the vascular barrier (Jiang et al., 2020). Exos from hypoxic human umbilical vein endothelial cells deliver miR-206 and miR-1-3p to inhibit neuronal RMRP, regulate the PI3K/Akt/mTOR/eNOS pathway to protect blood vessels, and alleviate neuronal ischemic injury (Zhong and Luo, 2021). Exercise-pretreated endothelial progenitor cell Exos target the ischemic penumbra, inhibit the p38 pathway, protect vascular tight junctions, restore local cerebral blood flow, and improve cerebral ischemic injury in patients with hypertension (Chen et al., 2026).

### 4.5 Balancing autophagy and endoplasmic reticulum stress

Autophagy exerts bidirectional regulatory effects: moderate autophagy clears damaged organelles and exerts a protective role, whereas excessive autophagy exacerbates neuronal death; Exos can precisely regulate the autophagy pathway to maintain homeostasis. Exos from hypoxia-reoxygenation-treated astrocytes highly express the Nampt protein, which activates the AMPK/mTOR pathway to induce neuroprotective autophagy; inhibiting Exos secretion or knocking down Nampt eliminates this protective effect (Deng et al., 2022). Exos from astrocytes subjected to ischaemic preconditioning carry circSHOC2, which binds to miR-7670-3p via the ceRNA mechanism to upregulate SIRT1, thereby promoting protective autophagy, blocking neuronal apoptosis, and alleviating ischaemic-hypoxic injury (Chen W. et al., 2020).

### 4.6 Promotion of angiogenesis and neural remodeling

Late-stage ischemia-induced angiogenesis, axonal regeneration, myelin repair, and synaptic remodeling are key components of long-term functional recovery in brain tissue. Various types of natural and engineered Exos can simultaneously regulate multiple pathways involved in angiogenesis and neural plasticity. Exos from adipose-derived stem cells (ADSCs) overexpressing miR-126 exhibit more pronounced anti-inflammatory, pro-angiogenic, and neurogenic effects than native Exos; native ADSC Exos can also enhance synaptic plasticity and mitigate infarct damage by delivering growth factors and functional miRNAs (Rau et al., 2025). Microglial Exos containing miR-212-5p silence PLXNA2, reduce infarct volume, and improve motor function, demonstrating their potential as both therapeutic agents and diagnostic biomarkers (Li et al., 2023). Leeches can regulate endothelial Exos to activate the HIFα-VEGF-DLL4-Notch1 pathway, thereby promoting pericyte proliferation, accelerating the maturation of new blood vessels in ischaemic areas, and restoring collateral circulation (Cao et al., 2025). BMSC Exos deliver the transcription factor Egr2 to activate SIRT6 and inhibit Notch1 signalling, thereby promoting endothelial angiogenesis and alleviating neuronal ischaemic damage (Xiao et al., 2023). miR-26a in human urine-derived stem cell Exos specifically inhibits HDAC6, activates the proliferation and differentiation of brain-derived neural stem cells, and initiates endogenous neurogenesis (Ling et al., 2020). miR-21-5p in BMSC Exos activates the VEGF/VEGFR2 and Ang-1/Tie2 pathways, promoting endothelial proliferation and tubular formation while increasing microvascular density in ischemic areas (Hu et al., 2022). Exos from brain endothelial cells can be transported retrograde along axons to the neuronal soma, where they upregulate pro-regenerative miRNAs and downregulate axon growth-inhibiting proteins, thereby inducing axonal outgrowth in cortical neurons; Exos from ischemic endothelial cells exhibit even stronger pro-regenerative activity (Zhang Y. et al., 2020). miR-128-3p in neural stem cell Exos specifically inhibits the BMP pathway, reverses fibrinogen-mediated differentiation arrest of oligodendrocyte precursor cells, and repairs demyelination damage following ischemia (Hou et al., 2023). A Sema3A inhibitor promotes the secretion of protective, prostaglandin D2 synthase-rich Exos by astrocytes, thereby regulating the neuronal Rnd1/R-Ras/Akt/GSK-3β pathway to breach the glial scar barrier and promote axonal regeneration around the infarct (Hira et al., 2018). Engineered MSC Exos with highly enriched miR-17–92 clusters downregulate PTEN, activate the PI3K/Akt/mTOR pathway, promote axonal regeneration and myelin remodeling, and improve long-term neurophysiological and motor function (Xin et al., 2021; Xin et al., 2017a). Rehabilitation exercise increases the brain accumulation of circulating Exos, promotes synaptogenesis, and maintains the integrity of the corticospinal tract; the combination of exercise and Exos intervention exhibits synergistic reparative effects (Li C. et al., 2021). miR-486 in BMSC-derived Exos specifically inhibits PTEN to activate the Akt pathway, promoting endothelial proliferation and migration and increasing microvascular density (Bao et al., 2024). Engineered MSC Exos overexpressing miR-133b stimulate astrocytes to secrete Exos that promote axonal regeneration, thereby enhancing neural plasticity in the peri-infarct region and improving long-term functional recovery (Xin et al., 2017b). The protective mechanisms of therapeutically administered exogenous exosomes are summarised in Table 2.

**TABLE 2:** Protective mechanisms of exogenously administered therapeutic Exos against cerebral ischemia–reperfusion injury.

Exosomes source	Key functional cargo	Signaling axis	Functional category	Main biological outcomes	Ref.
hUMSCs	CRYAB protein	CRYAB-ARRDC3-DRP1	Anti-ferroptosis, neuroprotection	Cross BBB via intranasal delivery; improve neurological function; inhibit Drp1-dependent mitochondrial fragmentation and neuronal ferroptosis	(ji et al., 2026)
ADSCs	miR-760-3p	miR-760-3p/CHAC1	Anti-ferroptosis, neuroprotection	Intranasal delivery enables brain accumulation; improves neurobehavioral function; inhibits neuronal ferroptosis by targeting CHAC1	(Wang et al., 2023a)
FNDC5-overexpressed BMSCs	Irisin	YAP/EGR1/ACSL4	Anti-ferroptosis, neuroprotection	Elevate neuronal viability; reduce infarct volume; improve neurological deficits; suppress YAP-EGR1-ACSL4-driven neuronal ferroptosis	(Fang et al., 2025)
Umbilical cord-mesenchymal stem cells (UC-MSCs)	circBBS2	circBBS2/miR-494/SLC7A11	Anti-ferroptosis, neuroprotection	Elevate neuronal viability; reduce infarct volume; repress neuronal ferroptosis via ceRNA regulatory mechanism	(Hong et al., 2023)
M2 microglia	lncRNA OIP5-AS1	OIP5-AS1/ITCH-TXNIP	Anti-pyroptosis, neuroprotection	Reduce infarct volume and brain edema; improve neurological scores; promote TXNIP ubiquitin-mediated degradation and suppress neuronal pyroptosis	(Li et al., 2024)
Melatonin-pretreated plasma Exos	Multiple differentially expressed miRNAs	TLR4/NF-κB	Anti-pyroptosis, anti-inflammation, neuroprotection	Reduce infarct volume and brain water content; improve neurological function; suppress NLRP3 inflammasome-dependent pyroptosis and neuroinflammation	(Wang et al., 2020a)
hNSCs	Unspecified endogenous cargo	PINK1/Parkin mitophagy	Mitophagy activation, anti-apoptosis, neuroprotection	Mitigate brain injury; improve behavioural function; activate mitophagy to repair mitochondrial dysfunction and reduce neuronal apoptosis	(Zhao et al., 2025a)
Neural progenitor cells	miR-100-5p	miR-100-5p/NOX4	Antioxidative stress, angiogenesis promotion, neuroprotection	Inhibit NOX4-driven ROS generation; reduce apoptosis; shrink infarct volume; improve neurological performance	(Zhao et al., 2025b)
M2-polarized macrophages	Unspecified endogenous cargo	Nrf2/HO-1	Antioxidative stress, anti-apoptosis, neuroprotection	Elevate neuronal viability; reduce oxidative injury and neuronal apoptosis; neuroprotective effect is partially abrogated by Nrf2 inhibitor ML385	(Xiao et al., 2022)
Endothelial progenitor cells (EPCs)	miR-210	Not specified	Antioxidative stress, anti-apoptosis, neuroprotection	Internalised by neurons; elevate neuronal miR-210; improve cell viability; suppress H/R-induced apoptosis and ROS overproduction	(Yerrapragada et al., 2022)
BMSCs	KLF4	KLF4-lncRNA-ZFAS1/FTO/Drp1 (m^6^A modification) axis	Mitochondrial homeostasis regulation, anti-oxidative stress, anti-apoptosis, neuroprotection	Improve neuronal viability; reduce oxidative stress, mitochondrial damage and apoptosis; shrink infarct area; KLF4 knockdown abolishes the protective effects	(Wang et al., 2023b)
BMSCs	miR-133a-3p	miR-133a-3p/DAPK2-Akt/mTOR axis	Autophagy inhibition, anti-apoptosis, neuroprotection	Restore neuronal proliferation; suppress OGD/R-triggered apoptosis and excessive autophagy; activate Akt/mTOR signalling; mitigate cerebral ischaemia-reperfusion injury *in vivo*	(Yang et al., 2023)
ADSCs	miR-22-3p	miR-22-3p/KDM6B/BMP2/BMF axis	Anti-apoptosis, neuroprotection	Mitigate cerebral I/R injury; improve neuronal viability; suppress neuronal apoptosis; inhibition of miR-22-3p weakens the protective effects	(Zhang et al., 2021)
BMSCs	lncRNA KLF3-AS1	KLF3-AS1/miR-206/USP22-Sirt1 axis	Anti-inflammation, anti-apoptosis, anti-oxidative stress, neuroprotection	Reduce infarct volume; improve neurological function; elevate cell viability; suppress apoptosis, inflammation and ROS accumulation; miR-206 overexpression or Sirt1 silencing reverses protective effects	(Xie et al., 2023)
Astrocytes	miR-190b	miR-190b/Atg7 axis	Autophagy inhibition, anti-apoptosis, neuroprotection	Delivered miR-190b to neurons; suppressed excessive autophagy via targeting Atg7; alleviated OGD-triggered neuronal apoptosis	(Pei et al., 2020)
hNSCs	Electroporation-loaded miR-125a-5p	miR-125a-5p/IKBKG-TLR4/NF-κB axis	Anti-neuroinflammation, microglia reprogramming, anti-apoptosis, neuroprotection	Promote M2 microglial polarization; reduce inflammatory cytokine release; suppress neuronal apoptosis; improve axonal ultrastructure and neurological function	(Liu et al., 2025b)
hUMSCs	HMGB1	HMGB1-TREM1-p38 MAPK/NF-κB axis	Anti-neuroinflammation, microglia reprogramming, neuroprotection	Intranasally delivered Exos accumulate in ischemic brain; shift microglia to anti-inflammatory phenotype; suppress microglial activation, migration and neuronal apoptosis; improve neurological outcomes	(Zhang et al., 2025a)
BMSCs	miR-21a-5p	miR-21a-5p/TLR2/NF-κB/MMP2/9 axis	BBB protection, anti-hemorrhagic transformation, neuroprotection	Elevate tight-junction protein expression; alleviate BBB leakage and hemorrhagic transformation; reduce infarct size; improve neurological recovery	(Yang et al., 2026)
Cerebral microvascular endothelial cells	Unspecified endogenous cargo	PDGF-PDGFRβ, Ang1/Ang2-Tie2	BBB protection, neuroprotection	Elevate tight-junction/basement-membrane protein levels; reduce BBB leakage; improve neurobehaviour; inhibitors of PDGFRβ/Tie2 abolish protective effects	(Sun et al., 2025b)
Ischemia-preconditioned astrocytes (IPASs)	circSHOC2	circSHOC2/miR-7670-3p/SIRT1 autophagy	Autophagy regulation, anti-apoptosis, neuroprotection	Inhibit neuronal apoptosis; modulate autophagy; mitigate cerebral ischemic injury; SIRT1 knockdown abrogates protective effects	(Chen et al., 2020b)
ADSCs	miR-126	miR-126-mediated multi-pathway	Anti-inflammation, anti-apoptosis, pro-angiogenesis, pro-neurogenesis	Improve neurological recovery; enhance angiogenesis and neurogenesis; inhibit microglial activation and neuronal apoptosis; superior efficacy compared to naïve ADSC-Exos	(Rau et al., 2025)
BMSCs	miR-21-5p	miR-21-5p/VEGF-VEGFR2, Ang-1-Tie-2 axis	Pro-angiogenesis, neuroprotection	Improve neurological function; reduce infarct volume; increase microvessel density; promote endothelial proliferation, migration and tube formation	(Hu et al., 2022)
NSCs	miR-128-3p	miR-128-3p/ACVR1-BMP	Promote OPC differentiation, remyelination, neuroprotection	Antagonize fibrinogen-triggered demyelination; boost OPC-to-oligodendrocyte differentiation; reduce infarct volume; improve neurological function	(Hou et al., 2023)
Human amniotic epithelial cells	FoxM1 transcription factor	FoxM1 signalling	Anti-inflammation, anti-oxidative stress, anti-apoptosis, neuroprotection	hAECs-Exos deliver FoxM1, suppress inflammation, oxidative stress and neuronal apoptosis; FoxM1 knockdown abolishes neuroprotective effects	(Wang et al., 2025)
Human serum	miR-340-5p	miR-340-5p/CD147 axis	Serum exosomal miR-340-5p directly targets and inhibits CD147, promotes BMEC angiogenesis after ischaemia	Serum Exos alleviate IS injury and promote angiogenesis; IS-exos show weaker protection due to miR-340-5p downregulation	(Xu et al., 2022)

## 5 Engineered exosomes for optimized brain-targeted delivery

Exos enter the brain through several pathways depending on administration routes and BBB status. For intravenous Exos under physiological conditions, brain endothelial transcytosis serves as the main route, including receptor-mediated transcytosis (TfR, LRP1, integrins, HSPGs), adsorptive transcytosis and macropinocytosis. Rab-family GTPases determine whether internalized Exos undergo trans-endothelial transport or lysosomal degradation. In IS, reduced tight-junction proteins (ZO-1, occludin, claudin-5) open a transient paracellular pathway analogous to the EPR effect; this leakage peaks within 72 h after ischemia and disappears with BBB repair, and is only functional in the acute phase. Intranasal Exos can bypass the BBB and reach the brain via olfactory-nerve and trigeminal-nerve-related perineural spaces. However, native Exos exhibit poor brain-targeting efficiency, which necessitates various engineering modification strategies.

Natural stem cell- and cell-derived Exos suffer from limitations such as limited brain-targeting efficiency, rapid clearance *in vivo*, weak drug-loading capacity, and poor batch-to-batch stability, which hinder their precise therapeutic efficacy in IS. Engineered Exos, constructed through pre-treatment interventions, surface-targeted modifications, genetic recombination of contents, drug loading, and novel carrier-combination strategies, can significantly enhance exosomal BBB permeability, lesion-targeted enrichment efficiency, and neuro-repair efficacy. This represents the core optimization direction for Exos-based targeted therapy in stroke. This chapter systematically summarises the technical characteristics, targeting mechanisms, and neuroprotective effects of various engineering strategies, as well as the application advantages and existing limitations of each modification approach.

### 5.1 Engineering of exosomes via pretreatment

Pre-treatment methods such as cellular hypoxia and cytokine stimulation can directly reshape the Exos secretion profile of parent cells, enriching protective nucleic acids and protein cargoes, thereby enhancing Exos neuro-repair activity without the need for complex *in vitro* modifications. Exos from hypoxic-pretreated mesenchymal stem cells are highly enriched in miR-214-3p; by specifically inhibiting PTEN and activating the Akt signaling pathway, they continuously modulate damage in the ischemic penumbra, significantly reducing cerebral infarct volume and improving neurological deficits; Silencing miR-214-3p completely abolished its neuroprotective effect, confirming that the miR-214-3p/PTEN/Akt axis is the core mechanism by which hypoxia-pretreated Exos exert neuroprotection (Wu et al., 2024). Furthermore, IFN-γ pretreatment can remodel the cargo composition of neural stem cell Exos, leading to the high expression of functional small non-coding RNAs, including miR-206, miR-133a-3p, and miR-3656, thereby significantly enhancing neuronal proliferation and anti-apoptotic capacity. Compared with conventional neural stem cell Exos, these Exos demonstrate superior effects in brain ischemia repair (Zhang G. et al., 2020). This type of pretreatment is simple to perform and highly safe; it can effectively enhance the endogenous reparative potential of Exos and serves as a classic, low-cost strategy for optimizing Exos function.

### 5.2 Strategies for targeted modification of exosomes surfaces

Modifying the Exos surface with brain-targeting peptides, specific antibodies, or functional ligands endows Exos with the ability to home to ischemic lesions, overcoming the limitations of diffuse targeting in native Exos and enabling precise delivery to neurons, microvascular endothelial cells, and microglia. The RVG brain-targeting peptide is the most commonly used modification component for stroke-derived Exos. RVG-miR21-sEVs, constructed based on the RVG peptide, can precisely target ischemic neurons; by regulating the PTEN/Akt/mTOR pathway, they inhibit OGD/R-induced excessive neuronal autophagy andmitochondrial damage, and apoptosis, thereby effectively reducing cerebral infarct volume and improving neurological deficits in mice (Li et al., 2026); RVG-Lamp2b-modified Exos can efficiently deliver miR-124 to ischemic brain tissue, promoting neural progenitor cell differentiation and activating endogenous neurogenesis in the brain (Yang et al., 2017). Furthermore, RVG-modified engineered Exos loaded with NGF can target the ischemic cortex, bidirectionally regulating anti-inflammatory microglial polarization and neural stem cell proliferation and differentiation, thereby achieving long-lasting neuroprotection (Yang et al., 2020).

cRGD peptide modification specifically recognizes the endothelium of ischemic-damaged blood vessels; cRGD-engineered Exos loaded with miR-210 can accumulate specifically at cerebral ischemic lesions, upregulate VEGF and CD34 expression to promote angiogenesis, and improve survival rates in animal stroke models (Zhang et al., 2019); cRGD-modified Exos loaded with curcumin can efficiently penetrate the lesion site, significantly suppressing local inflammatory responses and neuronal apoptosis, thereby providing a mature modification system for targeted drug delivery in stroke therapy (Tian et al., 2018). In addition to peptide modification, antibody-targeted and ligand-targeted approaches also demonstrate excellent precision: GAP43 monoclonal antibody-modified Exos loaded with quercetin can specifically recognize damaged neurons, activate the Nrf2/HO-1 antioxidant pathway to scavenge ROS, and alleviate cerebral ischemic injury (Guo et al., 2021a); Mannose-modified platelet-derived engineered Exos can achieve dual targeting of cerebral vascular endothelium and microglia by leveraging the ischemic homing properties of platelets and the targeted action of mannose receptors, thereby inhibiting M1-type microglial polarization and repairing the structure of the neurovascular unit (Liu et al., 2024).

### 5.3 Modification of exosomes contents: Overexpression of protective miRNAs and loading of small-molecule drugs

By genetically overexpressing functional nucleic acids and proteins, or by loading small-molecule drugs, active polysaccharides, and functional enzymes *in vitro*, the internal cargo of Exos can be directly modified to precisely enhance therapeutic effects, including anti-inflammatory, antioxidant, and regenerative properties. This is a core strategy for improving the specificity of Exos therapy. Regarding protective genetic modification, engineered MSC Exos overexpressing miR-132-3p can target the microvascular endothelium in ischemic brain tissue, activate the RASA1/RAS/PI3K/AKT pathway to inhibit endothelial oxidative stress and apoptosis, stabilize the BBB, and reduce infarct volume (Wang Zhenhua et al., 2024); miR-126-enriched engineered endothelial progenitor cell Exos can sustainably activate the VEGFR2 pathway, promoting angiogenesis and neural regeneration in ischemic areas, and significantly improving cerebral blood flow perfusion and long-term neurological function in a diabetic stroke model (Wang J. et al., 2020); meanwhile, endothelial Exos-derived miR-126 can also improve post-stroke cognitive function by regulating axonal myelin regeneration and macrophage polarization (Venkat et al., 2019). Dual-function genetically modified EXO-PD-L1-HGF-engineered Exos can target ischemic brain regions via the SDF-1α/CXCR4 axis, remodel the central and peripheral immune microenvironments, and activate the STAT3-FOXO3 pathway to promote neurogenesis and inhibit neuronal apoptosis (Lin et al., 2024).

In the field of drug and bioactive compound delivery, the quercetin-loaded targeted Exos developed by Yang Jialei’s team in 2025 can specifically eliminate p21^+^CD86^+^ senescent inflammatory microglia, block inflammatory cascades, and repair the BBB, thereby providing a novel strategy for anti-inflammatory stroke treatment (Yang et al., 2025c). The Exo-Lip complex—a combination of neural stem cell Exos and magnolia polysaccharide liposomes—developed by Chen Chunxia’s team in 2025, combines high BBB permeability with high drug-loading capacity. Activation of the AKT/Nrf2/HO-1 pathway suppresses neuroinflammation and lipid peroxidation, thereby achieving multifaceted, synergistic protection (Xie et al., 2025). The DNase 1-loaded M2 macrophage Exos prepared by the team of Guo Xing and Zhou Shaobing in 2024 can efficiently penetrate the BBB; by degrading NETs, inducing M2 polarization of microglia, and promoting vascular remodeling, they alleviate ischemia-reperfusion injury (Wang Zhenhua et al., 2024). Furthermore, BDNF-loaded neural stem cell Exos can significantly antagonize oxidative stress, inhibit microglial activation, and promote neural stem cell differentiation, with repair effects that are significantly superior to those of native Exos (Zhu et al., 2023) (Figure 6).

**FIGURE 6:**
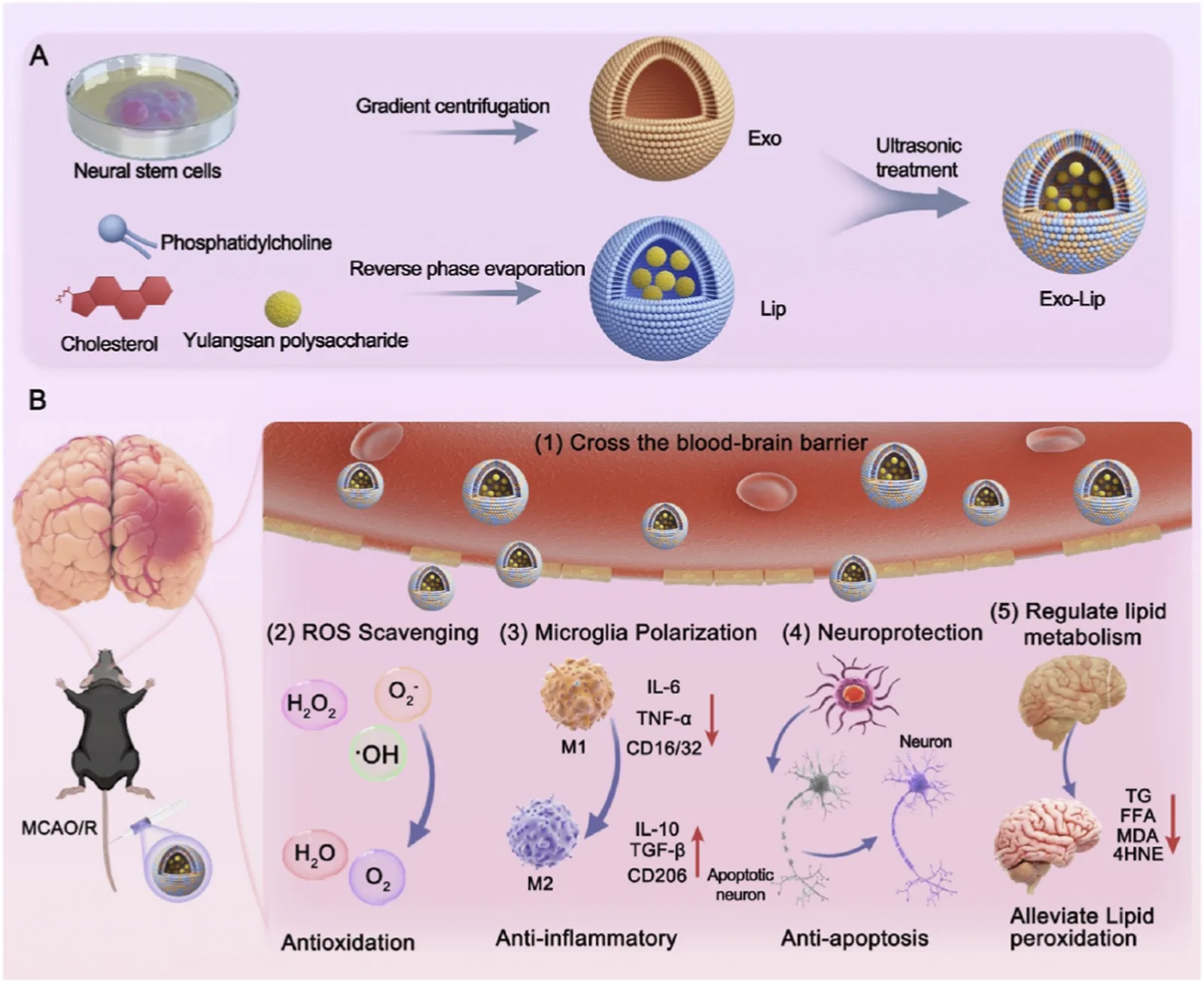
**(A)** Schematic diagram illustrating the fabrication workflow of Yulangsan polysaccharide_loaded Exo_Lip hybrid nanoparticles. **(B)** Schematic showing the in_vivo therapeutic mechanisms of Exo_Lip in the MCAO/R ischemic_stroke mouse model, including blood_brain_barrier penetration, ROS scavenging, microglia polarization modulation, neuroprotection and alleviation of lipid peroxidation injury. This platform integrates the BBB permeability and anti-inflammatory activity of Exos with the structural stability and drug-loading advantages of liposomes. By promoting M2 polarization of microglia, inhibiting the release of pro-inflammatory factors, and activating the AKT/Nrf2/HO-1 antioxidant signaling pathways, it effectively reduces cerebral infarct volume, alleviates lipid peroxidation damage, and restores motor and cognitive functions, demonstrating a synergistic neuroprotective effect (Xie et al., 2025).

### 5.4 Novel low-cost exosomes-like nanocarriers

To address the issues of short *in vivo* half-life and insufficient retention time at the lesion site, researchers have developed long-acting, stable Exos delivery systems using novel strategies such as drug-carrier conjugation, gel-based sustained release, and transnasal targeted delivery. Macrophage Exos and plasma Exos loaded with edaravone can significantly enhance drug bioavailability and brain-targeting efficiency. Through transferrin receptor-mediated transmembrane accumulation, they efficiently scavenge ROS and regulate M2 polarization of microglia, resulting in greater therapeutic efficacy and improved safety compared with drug therapy alone (Li F. et al., 2020; Guo et al., 2021b). Intranasally delivered RAGE-peptide-modified Exos can carry AMO181a-chol, specifically silencing miR-181a—which is highly expressed following ischemic injury—thereby upregulating the anti-apoptotic protein Bcl-2 and inhibiting inflammatory factors and neuronal apoptosis (Kim et al., 2021). Hyaluronic acid-adhering hydrogel-conjugated neural stem cell Exos enable long-term retention at the lesion site, continuously promoting cerebral angiogenesis and suppressing neuroinflammation, thereby improving motor and neurological function in stroke-affected mice over the long term (Gu et al., 2023); the injectable hydrogel-conjugated Exos system effectively prolongs drug retention time in brain tissue and significantly enhances the efficiency of Exos-mediated neural remodeling, making it a long-acting delivery platform with great translational potential (Zhang Q. et al., 2025). Cyanobacterial-derived Exos combine the advantages of spontaneous near-infrared fluorescence and the ability to cross the BBB, enabling label-free tracking of cerebral ischemic injury; they exert neuroprotective effects by blocking the ROS-NF-κB pathway and modulating lipid metabolism, and possess good biosafety, making them a natural nanocarrier with potential for large-scale application in the integrated diagnosis and treatment of stroke (Pan et al., 2026) (Figure 7). Representative pre‐clinical studies of engineered exosomes for brain‐targeted ischemic stroke therapy are presented in Table 3.

**FIGURE 7:**
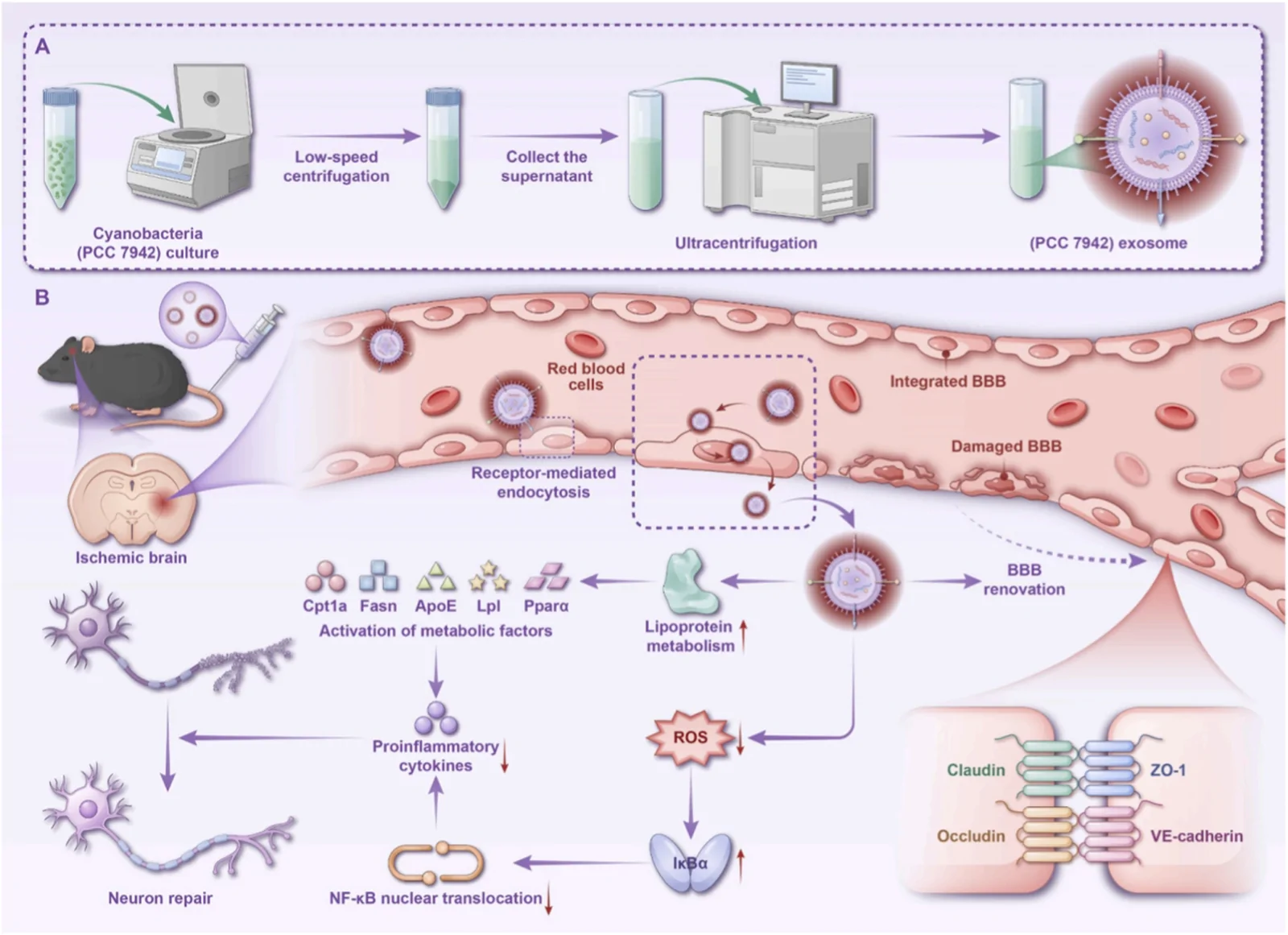
**(A)** Extraction and purification of exosomes derived from Synechococcus elongatus PCC 7942 (sExos). **(B)** Mechanisms of sExos_mediated ischemic stroke therapy. After administration, intrinsically fluorescent sExos cross the blood_brain barrier via receptor_mediated endocytosis, modulate lipid metabolism, suppress pro_inflammatory cytokines, reduce ROS, inhibit NF_κB signaling, and alleviate neuroinflammation to facilitate neuronal repair. A cyanobacteria-derived, naturally autofluorescent Exos has been developed for BBB-penetrant central nervous system (CNS) theranostics (Pan et al., 2026).

**TABLE 3:** Summary of representative studies on engineered Exos for brain-targeted therapy in IS.

Exos parent cell	Engineering modification strategy	Loaded/overexpressed cargo	Target signalling pathway	Core therapeutic effects	Main limitations	References
Mesenchymal stem cells	Hypoxia pre-conditioning	miR-214-3p	PTEN/Akt	Reduce infarct volume, improve neurological deficits	Low brain accumulation following systemic administration	(Wu et al., 2024)
Human neural stem cells	IFN-γ pre-conditioning	miR-206, miR-133a-3p, miR-3656	MAPK, PI3K-Akt, FoxO	Inhibit neuronal apoptosis, mitigate neuroinflammation and support neuronal survival	Non-standardized Exos purification protocols; unclear interaction mechanisms with immune cells	(Zhang et al., 2020b)
Bone marrow-derived MSCs	RVG-Lamp2b surface genetic modification; electroporation loading	miR-124	miR-124/Gli3-Stat3	Deliver miR-124 to ischemic cortex, enhance cortical neurogenesis and alleviate cerebral injury	Undefined cellular origin of newborn cortical neurons; targeting specificity requires further improvement	(Yang et al., 2017)
Bone marrow-derived MSCs	c (RGDyK) peptide surface conjugation; cholesterol-modified miRNA incubation loading	miR-210	miR-210/VEGF	Target cerebral ischemic lesions, promote endothelial angiogenesis and improve animal survival	Insufficient verification of downstream target genes; long-term biosafety remains unevaluated	(Zhang et al., 2019)
Adipose-derived stem cells	Native/genetic overexpression/surface modification	miR-126, miR-31, miR-21, growth factors, cytokines	VEGF/VEGFR2, PI3K/Akt, MAPK, TGF-β/Smad	Exert anti-inflammatory, antioxidant, pro-angiogenic and tissue-regenerative activities	Batch-dependent cargo heterogeneity; lack standardized purification; limited targeting efficiency; challenges for GMP-compliant manufacturing	(Rau et al., 2025)
Human astrocytes	DSPE-PEG-RVG peptide modification; ultrasound-assisted drug loading	SD208 (TGF-β1 receptor I inhibitor)	TGF-β1/Smad2/3	Reprogram astrocytes from neurotoxic A1 to reparative A2 phenotype, restore synaptic function, reduce infarct volume and ameliorate motor-cognitive deficits	Absence of human primary-cell validation; insufficient large-animal long-term efficacy data; unmonitored intracellular drug release kinetics	(Zhao et al., 2026b)
Neural stem cells	Hybrid fusion with Yulangsan polysaccharide-loaded liposomes (Exos-Lip)	Yulangsan polysaccharide	AKT/Nrf2/HO-1	Attenuate neuroinflammation and oxidative stress, restore lipid homeostasis, reduce infarct volume and rescue motor-cognitive function	Complicated preparation workflow for hybrid nanoparticles	(Xie et al., 2025)
CD206^+^ M2-like macrophages	Enzyme loading	DNase 1	NET-mediated inflammatory axis; M1/M2 microglial phenotypic switching	Cross the BBB, degrade neutrophil extracellular traps, induce M2 microglial polarization, facilitate BBB remodeling and neurological functional recovery	Short *in-vivo* half-life and low retention within lesion sites	(Wang et al., 2024b)
Bone marrow-derived MSCs	Lentiviral-mediated genetic overexpression in parental cells	CXCR4 protein	Wnt-3a/β-catenin	Promote endothelial proliferation and tube formation, exert anti-apoptotic effects on injured endothelium and enhance neurological recovery	Key functional cargo mediating angiogenesis remains uncharacterized	(Li et al., 2020a)
hTERT-ADSCs	PiggyBac transposon-based genetic engineering	PD-L1, HGF	STAT3-FOXO3 signalling cascade	Target peri-infarct brain regions, suppress T-cell-driven neuroinflammation, promote neurogenesis and achieve long-term motor-cognitive restoration	Further validation in large-animal models is required	(Lin et al., 2024)
HEK293F cells	Genetic fusion of microCH-Lamp2b; physical coincubation loading	Quercetin (senolytic agent)	p21-C/EBPβ-dependent SASP axis	Eliminate senescent pro-inflammatory microglia, lower SASP-related mediators, protect BBB integrity and improve sensorimotor performance	Potential off-target effects on CD86-positive antigen-presenting cells; no efficacy data from non-human primate or human studies	(Yang et al., 2025c)

## 6 Challenges and prospects for the clinical translation of exosomes in ischemic stroke

EVs come in several flavours—Exos, microvesicles, and apoptotic bodies—each arising through a different route and carrying different implications for stroke therapy. Exos (30–150 nm), budding from multivesicular bodies, have drawn the most interest: they are weakly immunogenic, can be loaded with diverse cargoes, cross the BBB reasonably well, and lend themselves to genetic tweaking. These assets make them the leading candidate among vesicles for treating CNS disorders. Microvesicles (100–1,000 nm), which pinch off directly from the plasma membrane, cross the BBB poorly and pose a hypothetical risk of clogging small vessels. Apoptotic bodies (500–5,000 nm) are simply cell fragments left over from apoptosis; they mostly propagate pathological signals and are rarely used in stroke research.

### 6.1 No industry-wide standards for making, purifying, and checking exosomes

Different labs use different isolation and purification methods; donor cells are cultured under varying conditions. The result is considerable batch-to-batch variability in what the vesicles contain and how they behave. There is currently no GMP-compliant production or quality-control pipeline designed specifically for stroke indications. This makes it hard to scale up manufacturing or ensure that one batch works like the last.

### 6.2 Getting exosomes to the brain: barriers, BBB interactions, and engineering fixes

Exos can cross the BBB by nature, but that does not mean systemic injection gets many of them into ischemic brain tissue. Most infused Exos end up trapped in the liver and spleen; only a tiny fraction ever reaches the damaged parenchyma. Once brain endothelial cells take them up, they are often sent to lysosomes for destruction. Rab-family proteins decide the vesicles' fate—transcytosis or degradation—and thus control how much cargo makes it into the brain. In the acute phase, tight junctions loosen, opening a temporary paracellular route rather than the EPR effect, so some Exos slip through. That window closes after about 72 h, too soon for the subacute or repair phases. On top of that, variation in donor cells adds another layer of inconsistency to *in vivo* results.

Context matters for how Exos affect BBB integrity. In healthy tissue, they move across the endothelium mainly via receptor-mediated transcytosis, with surface HSP70 helping the process without disrupting tight junctions. Under ischaemia–reperfusion, the picture splits. Damage-associated Exos from activated glia and foam cells carry Src, THBS1, and pro-inflammatory miRNAs that injure the microvasculature and make the leak worse. Protective ones, from stem cells or endothelial cells, do the opposite—they boost tight junction proteins and dampen MMP-2/9 activity, helping to repair the barrier.

A range of engineering tricks can improve targeting of brain lesions, but modifications are a double-edged sword. Too many targeting peptides on the surface, for instance, can actually hinder trans-BBB transport. Preconditioning donor cells with hypoxia or IFN-γ enriches protective content, yet it does not solve the fundamental problem of poor brain accumulation after systemic delivery. Tagging Exos with RVG or cRGD peptides directs them to neurons or damaged endothelium, respectively, and does increase local retention. Intranasal delivery, hydrogel depots, and Exos–liposome hybrids can bypass the liver and spleen or hold the vesicles at the injury site longer, but they come with their own headaches—complicated production, for one. The takeaway is that engineering should balance targeting, membrane integrity, and trans-endothelial ability, rather than chasing the highest ligand density.

### 6.3 Safety risks and unwanted effects

Nearly all safety data we have come from short rodent studies. Several concerns need a closer look before moving to patients.

First, vesicle preparations are never perfectly uniform. If they are contaminated with vesicles from M1 microglia or foam cells, those carry inflammatory miRNAs and proteins that could worsen neuroinflammation and ferroptosis. This underscores the need for rigorous purification and QC.

Second, in acute stroke the BBB is already leaky. High doses of Exos—or some engineered versions—might further destabilise the endothelium and, in theory, raise the chance of haemorrhage.

Third, most injected Exos end up in the liver and spleen. While Exos are not very immunogenic, allogeneic ones could still set off peripheral immune responses. For engineered Exos, there is also the worry of non-specific horizontal gene transfer. These vesicles shuttle a wide array of signalling molecules; whether any pro-proliferative or pro-fibrotic cargo could, over the long term, trigger tumours or excessive glial scarring is an open question—we lack large-animal or human data.

Fourth, if you give Exos intranasally or embed them in hydrogels, you have to consider mucosal irritation and foreign-body reactions to the material.

Importantly, most of these risks are still speculative or based on preclinical hints; we have little to no clinical evidence. Future trials should include safety endpoints as seriously as functional ones, covering both short- and long-term effects.

### 6.4 Human safety data are scarce

The list of potential risks is long, but human evidence is short. Almost everything comes from acute animal models. Immunotoxicity, chronic organ accumulation, and cancer risk—for both allogeneic and engineered Exos—have not been tested in humans. Other open questions: do heterogeneous vesicle populations inadvertently transfer pro-inflammatory payloads? Could Exos from cells with tumorigenic potential shuttle oncogenic molecules? Does high-dose intravenous infusion carry a real risk of microvascular blockage? Can genetic modifications cause off-target effects? None of these has been adequately examined in people.

### 6.5 Clinical infrastructure lags behind

We still lack standard dosing schedules for Exos therapeutics. Regulatory pathways and trial criteria for EV-based biologics are not yet mature, which holds back the move from bench to bedside.

## 7 Conclusion

Current clinical thrombolytic and thrombectomy approaches for IS suffer from limitations such as a narrow therapeutic window, reperfusion injury, and difficulty in crossing the BBB, and there is a lack of long-acting neuro-repair therapies. As natural EVs, Exos can carry bioactive substances such as miRNAs, proteins, and circular RNAs. They offer the advantages of low immunogenicity, the ability to cross the BBB, and multi-pathway regulation of tissue damage, making them highly promising carriers for cell-free stroke therapy.

Endogenous Exos in the ischemic microenvironment exert dual effects—both damaging and protective—while nucleic acids from peripheral blood Exos can serve as biomarkers for early stroke diagnosis and prognosis. Exos derived from exogenous stem cells, glial cells, and endothelial cells can inhibit oxidative stress, neuroinflammation, and various forms of programmed cell death; repair the BBB; and promote angiogenesis and neural remodeling, thereby effectively alleviating cerebral ischemia-reperfusion injury.

Through engineering approaches such as cell pretreatment, surface-targeted modification, genetic modification, and drug co-loading, the efficiency of Exos targeting and enrichment in brain tissue can be significantly enhanced, addressing the limitations of natural Exos, which are rapidly cleared from the body and fail to accumulate sufficiently at the lesion site. However, at this stage, this therapy still faces translational barriers, including a lack of preparation and quality control standards, limited *in vivo* delivery efficiency, insufficient data on long-term human safety, and incomplete clinical dosing regimens.

Overall, Exos hold great promise for both acute protection and long-term neural repair in IS. Future efforts should focus on refining standardized production and quality evaluation systems, thoroughly validating the safety of engineered Exos in humans, exploring synergistic treatment regimens—such as combination with thrombolysis and rehabilitation—and advancing Exos therapy from basic research to clinical application.

## Glossary

IS: ischemic stroke
Lip: liposome
BBB: blood–brain barrier
MDA: malondialdehyde
BSCB: blood-cerebrospinal fluid barrier
M1: pro-inflammatory microglial phenotype
miRNA: microRNA
M2: anti-inflammatory microglial phenotype
IκBα: inhibitor of κB α
NF-κB: nuclear factor κB
MVB: multivesicular body
Nrf2: nuclear factor erythroid 2-related factor 2
ROS: reactive oxygen species
OS: oxidative stress
ADSC: adipose-derived stem cells
TLR4: toll-like receptor 4
ARRDC3: arrestin domain-containing protein 3
VE-cadherin: vascular endothelial cadherin
BMSC: bone marrow mesenchymal stem cell
ZO-1: zonula occludens-1
DRP1: dynamin-related protein 1
Claudin-5: claudin 5 tight junction protein
ER: endoplasmic reticulum
Occludin: occludin tight junction protein
EVs/Exos/Exo: extracellular vesicles/Exos
IL-1β: interleukin 1 beta
Exo-Lip: Exo-liposome hybrid nanoparticle
IL-4: interleukin 4
GPX4: glutathione peroxidase 4
IL-6: interleukin 6
hNSC: human neural stem cell
IL-10: interleukin 10
hUMSC: human umbilical cord mesenchymal stem cell
TNF-α: tumor necrosis factor alpha
IKBKG: inhibitor of κB kinase γ
ILVs: intraluminal vesicles
MMP: mitochondrial membrane potential
IPASs: Ischemia-preconditioned astrocytes
